# Effect of verbal interference and response hand on hemisphere asymmetries in sad facial expression processing

**DOI:** 10.1371/journal.pone.0322504

**Published:** 2025-05-23

**Authors:** E. Darcy Burgund, Solana R. Cushing, Moura Saad

**Affiliations:** 1 Department of Psychology, Macalester College, Saint Paul, MN,; 2 Neuroscience Program, Macalester College, Saint Paul, MN; University of Birjand, IRAN, ISLAMIC REPUBLIC OF

## Abstract

A growing amount of evidence highlights a role for the left hemisphere in negative facial expression processing. The present study investigated the extent to which language contributes to this left hemisphere involvement by comparing performance during an emotion detection task presented to the left and right hemispheres using divided visual field under conditions of verbal interference (covertly rehearsing a 6-digit string for a subsequent memory) and no interference. Participants were college undergraduates with no known neurological or psychiatric conditions. Half used their right hand to respond and half used their left. In line with the hypothesis that language contributes to left hemisphere involvement in negative expression processing, participants who used their right hand to respond were more accurate with sad facial expressions when they were presented to the left hemisphere than the right during the no interference condition, but this left-hemisphere advantage disappeared during the verbal interference condition. Contrary to the hypothesis, participants who used their left hand to respond were more accurate with sad facial expressions when they were presented to the right hemisphere than when they were presented to the left, and this right-hemisphere advantage did not differ significantly between interference groups. Results highlight the influence of language as well as response hand on hemisphere asymmetries in facial expression processing and point towards areas for future research.

## Introduction

The ability to recognize others’ facial expressions is critical for successful interactions in a socially complex world. Research investigating hemisphere asymmetries in facial expression processing has demonstrated an important role for areas within the right hemisphere (e.g., [1–9]), especially for negative facial expression processing (e.g., [10–19]). This research is in line with general theories of hemisphere asymmetries in emotion processing, which argue that the right hemisphere is superior to the left (e.g., [20]) and that this is especially true for negative emotions (e.g., [21]). In contrast to this dominant perspective, a growing amount of evidence has highlighted situations in which the left hemisphere is dominant for negative facial expression processing [22–28]. The reasons for this left hemisphere involvement, however, are unclear.

One important factor may be the extent to which language is involved in facial expression processing, as left hemisphere regions are dominant for language in most people. The influence of language on facial expression processing has been demonstrated in a number of studies in which facial expression processing is affected by the presentation of emotion labels [29–32]. For example, Lindquist et al. [31] observed decreased facial expression categorization performance after labels describing the expressions (e.g., “sad”) had been made less accessible through satiation, while Nook et al. [32] observed increased facial expression recognition when expressions were paired with emotion labels. Moreover, the influence of language on facial expression processing has been tied to the left hemisphere. Burt and Hausmann [24] observed an effect of linguistic labels on facial expression processing for faces presented to the left hemisphere, but not for faces presented to the right hemisphere. Thus, it is possible that left hemisphere advantages in facial expression perception, especially those for negative facial expressions, are due to the influence of language on processing.

This hypothesis was tested in a recent study by Burgund [23] in which the six basic facial expressions (angry, disgusted, fearful, happy, sad, surprised) were presented to the left and right hemispheres using divided visual field. In Experiment 1, participants identified which facial expression was presented by pushing one of six keys corresponding to the six expressions. This task required participants to select linguistic labels for the expressions and thus required the use of language. In Experiment 2, participants simply detected whether facial expressions were emotional or neutral and were not required to identify which facial expression was presented. This detection task, therefore, did not require the use of language to the same extent that was required by the identification task. Results for fearful facial expressions were in line with the hypothesis that left hemisphere advantages in negative facial expression perception are due to the influence of language on processing—a left hemisphere advantage was observed during the identification task but not during the detection task. Notably, however, results for sad facial expressions were not, with equivalent left hemisphere advantages observed during the identification and detection tasks. Thus, language involvement may not be the entire explanation for left hemisphere advantages in negative facial expression processing.

Nonetheless, it is possible that language influenced processing during the facial emotion detection task even though it was not explicitly required. For example, participants could have mentally labeled the facial expressions (e.g., “sad”) and then decided whether the expression was emotional or not. If this happened, then the emotion detection task used in Burgund [23] may not have been as free of language influence as assumed, and language processing could have contributed to the left hemisphere advantage in this task. Indeed, several other studies observing left hemisphere advantages for negative facial expressions do not use tasks that require language explicitly [25–28], and therefore explicit language use cannot be a requirement if language influence is the explanation for left hemisphere advantages for negative facial expressions. As such, a manipulation that interferes with participants’ ability to use language during a non-linguistic facial expression task is needed in order to more fully examine this issue.

One way in which the influence of language on non-linguistic processing is interfered with is by having participants perform a verbal task at the same time as the primary task (see, e.g., [33]). This method assumes that performing the verbal task uses the language processing resources so that they cannot influence performance of the primary task. Memory-based tasks are a commonly used type of interference task in which participants are asked to covertly rehearse verbal material (often a string of 6–9 digits) while performing the primary task, and memory for the verbal material is tested subsequently (e.g., [34,35]). Thus, the present study used a memory-based verbal interference task to assess the influence of language on facial expression processing. In the primary task, participants detected whether facial expressions presented to the left or right hemisphere using divided visual-field were emotional or neutral, as in Burgund [23] and others [18,28]. Half of the participants performed this primary task while covertly rehearsing a 6-digit string for a subsequent memory test (verbal interference) and half simply performed the task (no interference). If language processing contributes to the left hemisphere advantage for negative facial expressions, then this advantage should be reduced in the verbal interference condition compared to the no interference condition. If language processing does not contribute to the left hemisphere advantage, then the advantage should not be reduced by interference.

The present study also examined the effect of response hand on hemisphere asymmetries in facial expression processing by having half of the participants in each interference group use their right (dominant) hand to respond and half use their left (non-dominant) hand to respond. Although response hand has an effect on visual hemisphere asymmetries in multiple contexts (e.g., [36–40]), previous studies using the divided-visual field emotion-detection task used in the present study have not observed response-hand effects [18,23]. However, these studies manipulated hand within participants, by switching response hand between blocks of trials, which decreased the number of trials per hand per participant, and may have reduced the studies’ sensitivity to a hand effect. As such, the between-participants manipulation of response hand in the present study was included in order to test this possibility. Critically, we did not expect response hand to have an effect.

## Method

### Ethics statement

The present experiment was approved by the Institutional Review Board at Macalester College (Approval number: 22010). Participants provided written consent in accordance with the guidelines established by the Code of Ethics of the World Medical Association (Declaration of Helsinki).

### Participants

Data collection occurred between April 12, 2022 and December 4, 2023. The initial participant sample consisted of 172 right-handed young adults with normal or corrected-to-normal vision recruited from Introduction to Psychology courses at Macalester College and the larger Macalester student body. However, since previous research indicates atypical hemisphere asymmetries in many common psychological and neurodevelopmental disorders (see, e.g., [41–43]), people who had been diagnosed with a neurological or psychological condition within the last five years and/or were currently taking medications to treat a diagnosed neurological or psychological condition (*N* = 52) were excluded.

The remaining 120 participants were distributed roughly evenly across the interference and the response hand conditions, as shown in Table 1. All participants were between 18–24 years old (*M* = 20.20, *SD* = 1.29). Approximately half (55%) identified as racially White; and participants of color identified as Asian (16%), mixed race (11%), Black (8%), Hispanic/Latinx (6%), Middle Eastern (2%), and South Asian (2%). The majority of participants (84%) spent most of their childhood in the United States of America. The next largest percentages were from Brazil and China with 2.5% each.

**Table 1:** Number, gender, and handedness for interference × response hand conditions.

	Response hand			
	Left hand		Right hand	
	Interference		Interference	
	None	Verbal	None	Verbal
*N*	27	28	31	34
Gender (f/m)	14/13	16/12	15/16	23/11
Handedness	.94 (.11)	.96 (.10)	.96 (.09)	.94 (.12)

‘*N*’ indicates the number of participants in each group. ‘Gender’ provides the number of female (f) and male (m) participants (there were not any nonbinary participants in the sample). ‘Handedness’ is the mean laterality quotient as determined by the Edinburgh Inventory [44], which produces scores from ‐1.0 (strongly left-handed) to 1.0 (strongly right-handed); parentheses indicate the standard deviation of the mean.

### Design

The experiment employed a 6 (expression: angry vs. disgusted vs. fearful vs. happy vs. sad vs. surprised) x 2 (hemisphere: left vs. right) x 2 (interference: none vs. verbal) x 2 (response hand: left hand vs. right hand) mixed-factorial design in which expression and hemisphere were within-participants independent variables, and interference and response hand were between-participants independent variables. A sensitivity analysis using G*Power 3.1 [45] revealed that, with an alpha of.05 and 80% power, 120 participants is sufficient to detect a small effect size of η_p_^2^ = .016 in this design. The dependent variable was sensitivity measured by d’ [46].

### Materials

Stimuli were photographs of 16 White individuals (8 female; 8 male) displaying 7 different expressions (angry, disgusted, fearful, happy, sad, surprised, and neutral) taken from the Radboud Faces Database [47]. Faces were depicted from the straight-on view gazing directly at the camera, and were gray-scaled, cropped to an oval shape that excluded the ears and hair, and presented on a white background. Each face subtended a visual angle of approximately 5.7 x 3.6˚ in vertical and horizontal dimensions, respectively, and was presented in the left or right visual field such that the center of each was approximately 5.4˚ from the center of the display and the inner edge was approximately 3.6˚ from the center. Participants placed their chins in a chinrest to keep their eyes approximately 20 inches from the computer screen. Response recording and stimulus presentation were controlled using PsyToolkit [48,49] on a Macintosh computer.

### Procedure

Participants began the study by completing a consent form that described the nature of the tasks they would perform. They were then randomly assigned to the no interference or the verbal interference condition. Both groups of participants then performed the emotion detection task in which they decided whether faces displayed emotional expressions or not. Each trial began with the presentation of a fixation cross (+) in the center of the screen for 1500 ms and was followed by the presentation of a face in the left or right visual field for 150 ms. After the face presentation, the center fixation cross remained on the screen until the participant pushed a key indicating their response and ended the trial. Within each interference condition, half of the participants used the index and middle fingers of their left hand to respond, with the ‘E’ key indicating ‘emotional’ and the ‘D’ key indicating ‘not emotional’, and half of the participants used the index and middle fingers their right hand to respond, with the ‘P’ key indicating ‘emotional’ and the ‘L’ key indicating ‘not emotional’. Participants were instructed to keep their eyes focused on the fixation cross throughout, and to respond as quickly and accurately as possible when each face appeared. Six practice trials were administered before the experimental trials began to ensure that participants understood the task instructions and familiarize them with the brief divided-visual field presentations and response keys. Experimental trials were administered in four blocks of 48 trials each, 24 emotional, with 4 for each of the 6 emotional facial expressions, and 24 not emotional, with half presented in the left visual field and half presented in the right. Stimuli within each block were presented in a pseudorandom order that was constrained such that no more than three of the same type of expression or visual field were presented in a row.

The difference between the no interference and verbal interference conditions was that participants in the verbal interference condition performed the above task while silently rehearsing a 6-digit number in order to recall it for a subsequent memory test. Participants in the verbal interference condition were instructed as follows:

“While you perform the facial expression task, you will be asked to remember a 6-digit number. An effective way to remember the number is to recite it over and over to yourself. The number will appear on the screen immediately before you begin the facial expression task, and you should remember it while you perform the task. At the end, you will be asked to enter the number.”

At the beginning of each block of trials, a 6-digit number appeared in the center of the screen. Participants looked at the number for as long as they wanted and pressed the spacebar when they were ready to begin the emotion detection task. After a block of 48 trials, participants were prompted to type in the 6-digit number. Then, when they were ready, they viewed another 6-digit number and completed the next block of trials. Participants completed 4 blocks of trials and remembered 4 different 6-digit numbers.

After completing 4 blocks of trials, all participants completed the Edinburgh Inventory to assess their handedness [44] and reported demographic information (age, gender, race, and countries lived in) as well as whether they had ever been diagnosed with a neurological or psychological condition, and if so, what condition and at what age, and whether they currently take medication to treat it. Participants were then debriefed, thanked, and compensated. The entire study took between 20–30 minutes.

## Results

Performance on the 6-digit number memory test in the verbal interference condition was assessed in terms of a strict and lenient coding of accuracy. In the strict coding of accuracy, a response was only considered correct when it matched the 6-digit number exactly (the correct digits in the correct order). Participants were moderately accurate using this strict coding, with mean accuracy of 85% (*SD* = 20%) in the left response-hand group and mean accuracy of 90% (*SD* = 15%) in the right response-hand group, which did not differ from each other, *t*(60) = 1.10, *p* = .274, *d* = .142, 95% CI [-.112,.396]. In the lenient coding of accuracy, a response was considered correct if 5 of the 6 digits were correct and in the correct order, or if all 6 digits were correct but two were reversed in order. With this more lenient coding of accuracy, participants in the left response-hand group had a mean accuracy of 93% (*SD* = 15%) and participants in the right response-hand group had a mean accuracy of 94% (*SD* = 12%). These means did not differ, *t*(60) =.362, *p* = .718, *d* = .047, 95% CI [-.206,.300].

**Fig 1:**
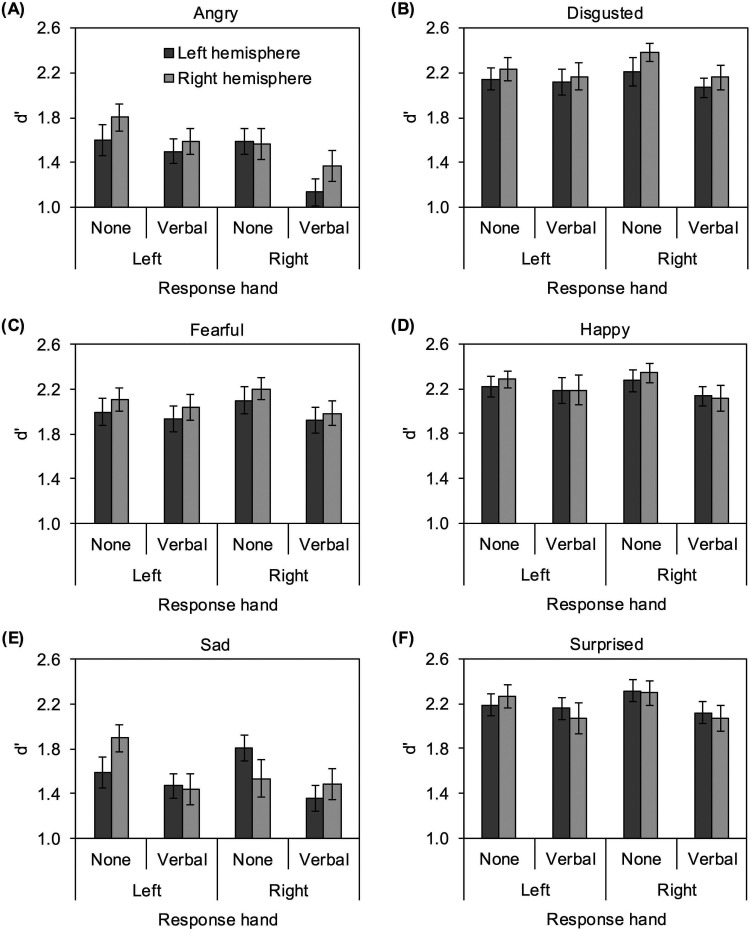
Mean d’ scores displayed as a function of expression, hemisphere, interference, and response hand. Vertical bars indicate standard error of the mean.

Performance on the emotion detection task was assessed in terms of d’, calculated as the difference between the normalized proportion of hits (correct response that a face was emotional) minus the normalized proportion of false alarms (incorrect response that a face was emotional). Because values of d’ are undefined when hit or false alarm rates are 1 or 0, proportions of 0 were converted to 1/(2*N*) and proportions of 1 were converted to 1-(1/(2*N*)), with *N* referring to the number of trials contributing to the proportion.

d’ scores were analyzed in a 6 (expression: angry vs. disgusted vs. fearful vs. happy vs. sad vs. surprised) x 2 (hemisphere: left vs. right) x 2 (interference: none vs. verbal) x 2 (response hand: left hand vs. right hand) repeated-measures analysis of variance (ANOVA) in which expression and hemisphere were within-participants independent variables, and interference and response hand were between-participants independent variables. Results are shown in Fig 1.

The main effect of interference was significant, *F*(1, 116) = 4.18, *p* = .043, η_p_^2^ = .035, 95% CI [-.032,.101]. d’ scores were higher in the no interference condition (*M* = 2.04, *SD* = .452) than the verbal interference condition (*M* = 1.86, *SD* = .466). The main effect of expression was also significant, *F*(5, 580) = 115.64, *p* < .001, η_p_^2^ = .166, 95% CI [.106,.227]. d’ scores were highest for happy (*M* = 2.22, *SD* = .504), disgusted (*M* = 2.19, *SD* = .499), and surprised (*M* = 2.18, *SD* = .533) expressions, which did not differ, happy vs. disgusted: *t*(119) = 1.41, *p* = .161, *d* = .129, 95% CI [-.051,.310]; happy vs. surprised: *t*(119) = 1.53, *p* = .130, *d* = .140, 95% CI [-.040,.321]; disgus*t*ed vs. surprised: *t*(119) =.150, *p* = .881, *d* = .014, 95% CI [-.166,.193]. d’ scores for fearful expressions were lower (*M* = 2.04, *SD* = .535), fearful vs. happy: *t*(119) = 5.37, *p* < .001, *d* = .492, 95% CI [.302,.683]; fearful vs. disgus*t*ed: *t*(119) = 4.62, *p* < .001, *d* = .424, 95% CI [.263,.611]; fearful vs. surprised: *t*(119) = 5.15, *p* < .001, *d* = .472, 95% CI [.283,.662]. d’ scores for angry (*M* = 1.50, *SD* = .608) and sad (*M* = 1.56, *SD* = .633) expressions were lower, angry vs. fearful: *t*(119) = 11.13, *p* < .001, *d* = 1.02, 95% CI [.799, 1.24]; sad vs. fearful: *t*(119) = 10.52, *p* < .001, *d* = .964, 95% CI [.747, 1.18], and did no*t* differ from each other, *t*(119) = 1.36, *p* = .176, *d* = .125, 95% CI [-.056,.305]. The expression x response hand interac*t*ion, *F*(5, 580) = 2.48, *p* = .031, η_p_^2^ = .004, 95% CI [-.006,.015], and the qualifying four-way interaction of expression x hemisphere x in*t*erference x response hand, *F*(5, 580) = 3.14, *p* = .008, η_p_^2^ = .005, 95% CI [-.007,.017], were also significant (all other *p*s > .107).

The four-way interaction was explored further by examining the three-way interaction of hemisphere x interference x response hand separately for each expression (see Table 2). The only expressions that exhibited significant effects in these analyses were angry and sad. For angry expressions (see Fig 1A), d’ scores were higher in the no interference condition (*M* = 1.64, *SD* = .588) than the verbal interference condition (*M* = 1.38, *SD* = .606), *F*(1, 116) = 5.01, *p* = .027, η_p_^2^ = .041, 95% CI [-.031,.114], for the main effect of interference condition. Similarly, for sad expressions (see Fig 1E), d’ scores were higher in the no interference condition (*M* = 1.70, *SD* = .642) than the verbal interference condition (*M* = 1.43, *SD* = .601), *F*(1, 116) = 5.47, *p* = .021, η_p_^2^ = .045, 95% CI [-.030,.121], for the main effect of interference condition.

**Table 2:** *F* values for effects from analysis of d’ in hemisphere x interference x response hand ANOVA for each expression.

Expression						
*Effect*	Angry	Disgusted	Fearful	Happy	Sad	Surprised
Hemisphere	3.90	3.68	2.89	.488	.252	.131
Interference	5.01*	1.53	1.80	1.72	5.47*	2.74
Response hand	3.68	.160	.124	.000	.217	.077
Hemisphere x interference	.321	.349	.062	.800	.047	.982
Hemisphere x response hand	.099	.364	.056	.014	2.59	.089
Hemisphere x interference x response hand	2.12	.036	.023	.035	8.13**	.523

* = *p* <.050; ** = *p* <.010.

Critically, the hemisphere x interference x response hand interaction was also significant for sad expressions, *F*(1, 116) = 8.13, *p* = .005, η_p_^2^ = .065, 95% CI [-.025,.156]. This three-way interaction was probed by examining the two-way interaction of hemisphere x interference condition separately for each response hand group. This two-way interaction was significant in participants who used their right hand to respond, *F*(1, 63) = 5.82, *p* = .019, η_p_^2^ = .085, 95% CI [.004,.247], with higher d’ scores for faces presented to the left hemisphere (*M* = 1.80, *SD* = .637) than the right (*M* = 1.53, *SD* = .926) in the no interference condition, *t*(30) = 2.13, *p* = .042, *d* = .389, 95% CI [.018,.760], and no difference between the left (*M* = 1.36, *SD* = .654) and right (*M* = 1.48, *SD* = .797) hemispheres in the verbal interference condition, *t*(33) = 1.19, *p* = .241, *d* = .207, 95% CI [-.138,.552] (see right side of Fig 1E). The two-way interaction of hemisphere x interference condition was not significant in participants who used their left hand to respond, *F*(1, 53) = 2.81, *p* = .100, η_p_^2^ = .050, 95% CI [.000,.212], however post-hoc *t* tests revealed higher d’ scores for faces presented to the right hemisphere (*M* = 1.89, *SD* = .614) than the left (*M* = 1.59, *SD* = .725) in the no interference condition, *t*(26) = 2.17, *p* = .040, *d* = .426, 95% CI [.024,.827], and no difference between the left (*M* = 1.47, *SD* = .579) and right (*M* = 1.44, *SD* = .726) hemispheres in the verbal interference condition, *t*(27) =.233, *p* = .818, *d* = .045, 95% CI [-.333,.422] (see left side of Fig 1E).

Response times for correct responses were analyzed similarly to d’ scores in a 6 (expression: angry vs. disgusted vs. fearful vs. happy vs. sad vs. surprised) x 2 (hemisphere: left vs. right) x 2 (interference: none vs. verbal) x 2 (response hand: left hand vs. right hand) repeated-measures ANOVA in which expression and hemisphere were within-participants independent variables, and interference and response hand were between-participants independent variables. Prior to analysis, response times that were longer than 3 standard deviations from the mean (1.8% of correct responses) were removed, and cells for which there were no remaining response times (.35%) were replaced with the grand mean. Results are provided in Table 3.

**Table 3:** Mean response times as a function of expression, hemisphere, interference, and response hand.

*Expression*	Response hand							
	Left hand				Right hand			
	Interference				Interference			
	None		Verbal		None		Verbal	
	Left	Right	Left	Right	Left	Right	Left	Right
Angry	775 (113)	804 (201)	822 (128)	859 (141)	842 (201)	859 (179)	850 (219)	850 (227)
Disgusted	662 (107)	683 (117)	727 (102)	717 (116)	689 (105)	693 (116)	718 (163)	709 (155)
Fearful	703 (137)	704 (128)	751 (92)	760 (125)	743 (128)	770 (125)	740 (145)	757 (127)
Happy	651 (127)	626 (105)	695 (131)	675 (104)	634 (79)	649 (80)	640 (131)	682 (143)
Sad	821 (182)	813 (182)	826 (103)	858 (179)	811 (170)	832 (161)	817 (221)	834 (136)
Surprised	671 (106)	673 (127)	725 (119)	700 (102)	678 (109)	701 (105)	688 (135)	716 (163)

‘Left’ indicates left-hemisphere presentations; ‘right’ indicates right-hemisphere presentations. Times are provided in milliseconds. Parentheses indicate standard deviation of the mean.

The analysis of response time revealed a main effect of expression, *F*(5, 580) = 126.31, *p* < .001, η_p_^2^ = .179, 95% CI [.116,.241]. Response times (ms) were fastest for happy expressions (*M* = 656, *SD* = 108), followed by surprised (*M* = 694, *SD* = 112), *t*(119) = 6.23, *p* < .001, *d* = .571, 95% CI [.377,.765], and disgus*t*ed expressions (*M* = 700, *SD* = 119), *t*(119) = 6.89, *p* < .001, *d* = .632, 95% CI [.435,.828], which did not differ, *t*(119) =.971, *p* = .333, *d* = .089, 95% CI [.091,.269]. Response times for fearful expressions (*M* = 742, *SD* = 114) were longer than *t*hose for surprised, *t*(119) = 6.90, *p* < .001, *d* = .633, 95% CI [.436,.829], and disgusted expressions, *t*(119) = 5.87, *p* < .001, *d* = .538, 95% CI [.346,.730]. Finally, response *t*imes for sad (*M* = 826, *SD* = 147) and angry expressions (*M* = 834, *SD* = 161) were longer than those for fearful expressions, sad vs. fearful: *t*(119) = 8.00, *p* < .001, *d* = .733, 95% CI [.531,.936]; angry vs. fearful: *t*(119) = 8.19, *p* < .001, *d* = .751, 95% CI [.547,.954], and did not differ from each other, *t*(119) =.762, *p* = .448, *d* = .070, 95% CI [-.110,.250].

The main effect of hemisphere was marginally significant, *F*(1, 116) = 3.65, *p* = .059, η_p_^2^ = .031, 95% CI [-.032,.093], with a trend towards faster response times in the right hemisphere (*M* = 737, *SD* = 112) than the left (*M* = 748, *SD* = 117). No other effects from the analysis of response times approach significance, all *p*s > .107.

## Discussion

While much previous research investigating hemisphere asymmetries in facial expression processing observes right hemisphere advantages for processing negative facial expressions (e.g., [10–19]), a growing amount of evidence highlights a role for the left hemisphere [22–28]. The present study investigated the extent to which language contributes to this left hemisphere involvement by comparing performance during an emotion detection task presented to the left and right hemispheres under conditions of verbal interference (covertly rehearsing a 6-digit string for a subsequent memory) or no interference. We predicted that, if the left hemisphere advantage for negative facial expressions is due to language contributions, it should be reduced in the verbal interference condition compared to the no interference condition. Critically, half of the participants within the no interference and verbal interference groups used their right hand to respond and half used their left hand. For sad facial expressions, results from participants who used their right hand to respond were in line with our prediction but results from those who used their left hand were not. Hemisphere asymmetries were not observed for the other facial expressions. Results are discussed below.

As stated above, results from participants who used their right hand to respond to sad facial expressions support the idea that left hemisphere involvement in negative facial expression processing is due to contributions from language (see, e.g., [23,24]). During the no interference condition, when language processes were free to contribute to facial expression processing, a left hemisphere advantage was observed for sad expressions. This advantage disappeared, however, during the verbal interference condition when language contributions were disrupted. As such, results provide an explanation for previous findings of left hemisphere advantages for negative facial expression processing during non-linguistic tasks (e.g., [23,25–28]) by suggesting that language may have contributed to processing despite not being required.

Importantly, participants who used their left hand to respond exhibited a different pattern of results. For these participants, a right hemisphere advantage was observed for sad expressions during the no interference condition, and this effect did not differ from that in the verbal interference condition, although the effect of hemisphere was not significant during verbal interference. This right hemisphere advantage for sad expressions replicates the right hemisphere advantage for negative facial expressions observed in many studies (e.g., [10–19]), however, the effect of response hand on the direction of hemisphere asymmetry for sad expressions was surprising. As noted in the Introduction, previous studies using the divided-visual field emotion-detection task used in the present study have not observed an effect of response hand [18,23] and the goal of including the response-hand variable in the present study was to rule out the existence of a response-hand effect in a potentially more powerful design. Critically, we did not achieve this goal, and thus, the possibility that response hand influences hemisphere asymmetries in facial expression processing is supported.

The effect of response hand on visual hemisphere asymmetries is not well understood. Some research suggests that performance is negatively impacted when the visual stimulus and motor response are processed in the same hemisphere (e.g., right visual field and right hand) compared to when they are processed in different hemispheres [37,39], while other research suggests a cooperative relationship in which performance is improved when the visual stimulus and motor response are processed in the same hemisphere [36]. Results from the present study are more in line with the cooperative perspective. Performance for sad facial expressions was better in the left than right hemisphere when motor responses were controlled by the left hemisphere (right hand), and better in the right than left hemisphere when motor responses were controlled by the right hemisphere (left hand). Clearly more research will be needed to understand this relationship, however the present research highlights the relevance of this relationship to facial expression processing.

Notably, sad expressions were the only expressions of the 6 tested that exhibited a hemisphere asymmetry. One reason for this could be that existing hemisphere asymmetries for the other facial expressions were obscured due to ceiling effects. Indeed, performance on sad expressions was lower than performance on all other expressions (except angry) as indicated by the main effect of expression in d’ scores and response times. However, ceiling effects cannot be the entire explanation for the lack of hemisphere asymmetries in the other expressions because, as noted, performance on angry expressions was also lower than the others and equivalent to sad expressions. As such, it is possible that sad facial expressions are unique in their engagement of regions within the left hemisphere during non-linguistic tasks.

A growing amount of evidence supports this possibility. As described in the Introduction, Burgund [23] observed a left hemisphere advantage for both sad and fearful facial expressions during an expression labeling task, but only sad expressions continued to exhibit this advantage when the requirement for linguistic labels was removed during an emotion detection task. In another study, Matt et al. [25] observed a greater EEG signal over occipital-temporal sites for sad facial expressions than fearful or happy expressions, especially in the left hemisphere. Similarly, Jia et al. [50] observed a greater N170 over parietal-occipital sites for infant faces with sad expressions compared to those with happy or neutral expressions, and for female participants, this effect was more pronounced in the left hemisphere than the right. Finally, in a meta-analysis including 141 fMRI studies of facial expression processing, Xu et al. [51] observed a hemisphere asymmetry in which activity for sad facial expressions was greater in the left ventromedial prefrontal cortex (vmPFC) than the right, while activity for fearful, happy, and disgusted facial expressions was greater in the right vmPFC than the left. The present study adds to this expanding body of work distinguishing sad expressions from other facial expressions in terms of left hemisphere engagement.

Critically, we attribute the left hemisphere involvement in sad facial expression processing to contributions from language, as this advantage was eliminated during the verbal interference condition when language contributions were disrupted. It is clear that the verbal interference task was “interfering” as d’ scores were lower in the verbal interference condition than the no interference condition overall, as shown by the main effect of interference condition, and for sad (and angry) expressions separately (see Table 2). It is also clear that participants in the verbal interference condition were performing the task of remembering the 6-digit number because their performance on the number memory test was high (> 93% with the lenient scoring criteria). What is unclear is whether the effect of the verbal interference task on hemisphere asymmetries was due to the verbal nature of the task or more simply due to the demands of performing a secondary task. Previous research has observed decreased right hemisphere involvement in facial expression processing when a non-verbal interference task is performed compared to control [52]. This is similar to the decreased right hemisphere performance in the verbal compared to no interference conditions for sad expressions in the present study when participants used their left hand, but contrasts with the lack of the same effect when participants used their right hand highlighting the difference between the verbal interference task used in the present study and a non-verbal interference task. Nonetheless, future studies should compare hemisphere asymmetries for facial expressions with no interference to those observed during verbal and non-verbal interference tasks in order to isolate the effect of language more definitively.

## Conclusion

In conclusion, the present study investigated the effect of verbal interference on hemisphere asymmetries in a non-linguistic facial emotion detection task. Results from participants who used their right hand to respond supported the hypothesis that verbal interference reduces the left hemisphere advantage for negative facial expressions, in particular sad facial expressions. Results from participants who used their left hand to respond did not support this hypothesis, highlighting the influence of response hand on hemisphere asymmetries in facial expression processing. Future research should investigate the effect of response hand further, as well as probe the extent to which effects of interference are due to language specifically or more general processing demands.
